# Correction: The transforming growth factor beta signaling pathway is critical for the formation of CD4 T follicular helper cells and isotype-switched antibody responses in the lung mucosa

**DOI:** 10.7554/eLife.06900

**Published:** 2015-02-12

**Authors:** Heather D Marshall, John P Ray, Brian J Laidlaw, Nianzhi Zhang, Dipika Gawande, Matthew M Staron, Joe Craft, Susan M Kaech

Marshall HD, Ray JP, Laidlaw BJ, Zhang N, Gawande D, Staron MM, Craft J, Kaech SM. 2015. The transforming growth factor beta signaling pathway is critical for the formation of CD4 T follicular helper cells and isotype-switched antibody responses in the lung mucosa. *eLife* **4**:e04851. doi: 10.7554/eLife.04851

Published 8 January 2015

TGF-β was incorrectly spelt out as ‘tumor growth factor beta’ both in the title and in the Digest. The correct title is: The transforming growth factor beta signaling pathway is critical for the formation of CD4 T follicular helper cells and isotype-switched antibody responses in the lung mucosa. The title and Digest have been corrected accordingly.

